# Prospective study of epilepsy with generalized tonic–clonic seizures alone: Clinical features, response to treatment, and likelihood of medication withdrawal

**DOI:** 10.1002/epi4.12981

**Published:** 2024-05-31

**Authors:** Fatima Jaafar, Jaafar Wazne, Ghassan Hmaimess, Wassim Nasreddine, Ayman Beydoun, AbdelRahman Shatila, Ahmad Beydoun

**Affiliations:** ^1^ American University of Beirut Medical Center Beirut Lebanon; ^2^ Rafic Hariri University Hospital Beirut Lebanon; ^3^ St George Hospital Medical University Center, University of Balamand Beirut Lebanon; ^4^ Lebanese American University Beirut Lebanon

**Keywords:** antiseizure medication, electroencephalography, idiopathic generalized epilepsy, prognostic factors, recurrence rate, withdrawal

## Abstract

**Objective:**

This prospective study aimed to delineate the demographics, natural progression, and treatment response of patients newly diagnosed with epilepsy with generalized tonic–clonic seizures alone (EGTCA). Furthermore, our objective includes assessing the seizure recurrence rate post antiseizure medication (ASM) discontinuation within this cohort, alongside exploring predictive factors for seizure relapse.

**Methods:**

The study cohort, derived from an ongoing, prospective, multicenter investigation on children and adults with new‐onset unprovoked seizures, included consecutive patients enrolled between March 2010 and March 2020, and meeting mandatory ILAE criteria for EGTCA diagnosis. Participants underwent a 3‐h sleep‐deprived video‐EEG recording along with an epilepsy protocol brain magnetic resonance imaging (MRI) with repeat EEG at each follow‐up. Cumulative time‐dependent probabilities of seizure recurrence were calculated using Kaplan–Meier survival analysis. Logistic regression identified variables associated with seizure recurrence following ASM taper.

**Results:**

Eighty‐nine patients with a median age of 16 years were included, constituting 31% of those diagnosed with an idiopathic generalized epilepsy. Regarding the circadian distribution of seizures, 59.6% of patients exclusively experienced diurnal seizures, 12.4% exclusively nocturnal, and 28.1% experienced both diurnal and nocturnal seizures. Generalized spike–wave discharges (GSWD) were present in the initial EEG of 88% of patients. A GTC recurred in 14% of patients treated with ASM compared with 73% of untreated patients (*p* < 0.00001). ASM discontinuation was attempted in 50 patients after a median treatment duration of 3 years, with 44% experiencing a recurrence. Patient‐initiated taper and a mixed circadian seizure pattern independently predicted a higher likelihood of recurrence post‐ASM discontinuation.

**Significance:**

Our findings underscore the importance of prompt treatment upon the diagnosis of EGTCA. Notably, lifelong treatment may not be imperative; patients seizure‐free for at least 2 years, with the absence of GSWD on EEG, often maintained seizure freedom after ASM withdrawal, especially with physician‐initiated tapering.

**Plain Language Summary:**

Seizures in individuals diagnosed with “epilepsy with generalized tonic‐clonic seizures alone” (EGTCA) typically start during adolescence and often respond well to antiseizure medications. An electroencephalogram, which measure brain waves, will show abnormal discharges in most patients with EGTCA. Lifelong treatment with antiseizure medication is not necessary for everyone with EGTCA; approximately, 40% can successfully stop treatment without facing seizure recurrence. Patients who stop medication on their own have a higher risk of seizures returning compared with those who undergo cessation under a doctor's supervision.

**Key points**

- Onset of seizures in EGTCA predominantly occurs during the second decade, and these seizures are usually responsive to ASM treatment.
- A 3‐h sleep‐deprived EEG revealed generalized spike–wave discharges in 88% of patients subsequently diagnosed with EGTCA.
- Lifelong treatment may not be mandatory for EGTCA, as successful tapering from ASM is achievable in 40% of patients.
- Patient‐initiated taper and a mixed circadian pattern of seizures predict a higher likelihood of seizure recurrence after ASM discontinuation.

## 1 INTRODUCTION

Epilepsy with generalized tonic–clonic seizures alone (EGTCA), formerly known as epilepsy with grand mal seizures on awakening, was initially described by Janz in 1953.^1^ This condition was reported to be characterized by generalized tonic–clonic (GTC) seizures occurring exclusively or predominantly upon awakening and during the evening relaxation period.^1^, ^2^ Recognized as an epilepsy syndrome by the International League Against Epilepsy (ILAE) in 1989, it was labeled ‘idiopathic generalized epilepsy with GTCs on awakening’.^2^ In 2001, an ILAE Task Force broadened this syndrome to include random and nocturnal GTCS.^3^ The latest revision of the ILAE classification of epilepsy syndromes in 2022 designates EGTCA as one of the four idiopathic generalized epilepsies (IGEs) syndromes^4^ and provides updated diagnostic criteria, including normal development, normal neurological examination and neuroimaging, exclusive GTCs seizures, seizure onset between 5 and 40 years, and presence of fast generalized spike or polyspike–wave discharges (GSWD) with a normal background on electroencephalography (EEG).^4^

Despite constituting up to 40% of IGE cases,^5^ epidemiologic data on EGTCA are limited and often unreliable due to inconsistencies in definitions related to circadian rhythm, seizure types, and age thresholds at seizure onset. Challenges in precisely defining EGTCA have hindered the establishment of prognostic and outcome measures. Notably, clear guidelines regarding the appropriate and safe timing of antiseizure medication (ASM) withdrawal in this population are lacking, given the uncertainty surrounding the prognosis post‐withdrawal.

This prospective study aimed to delineate the demographics, natural history, and treatment response of individuals newly diagnosed with EGTCA according to the latest ILAE criteria.^4^ Additionally, we seek to determine the recurrence rate after ASM discontinuation in this patient population and evaluate predictive factors for seizure relapse.

## 2 METHODS

### 2.1 Study design

The cohort for this study was derived from an ongoing prospective investigation conducted on patients with new‐onset unprovoked seizures in Lebanon (NOEL trial). This is a multicenter study centralized at the American University of Beirut Medical Center (AUBMC) and conducted in collaboration with the Lebanese Chapter of the ILAE. Adult and pediatric neurologists from all six governorates of Lebanon refer their patients with newly diagnosed seizures to the AUBMC, where a comprehensive clinical assessment and extensive workup are performed.

Initial visits include a comprehensive history with a detailed description of the seizure semiology obtained from patients and/or eyewitnesses and a complete examination. As per protocol, all patients undergo a 3‐h sleep‐deprived video‐EEG recording along with an epilepsy protocol brain magnetic resonance imaging (MRI). Follow‐ups include telephone consultations and yearly visits with repeat EEG studies, with more frequent visits scheduled in cases of seizure recurrence or adverse events. Information regarding seizure frequency, changes in drug therapy or posology and adverse events is systematically documented. Compliance is monitored by querying caregivers/patients about ASM administration. Regular serum levels' monitoring is conducted for valproate, phenytoin, or phenobarbital; however, newer ASM levels are rarely monitored due to local facility unavailability and high costs.

During the initial visit, patients are also asked about the presence of epilepsy risk factors. For this study, a positive family history of epilepsy is assigned to patients with affected first‐degree relatives (parents, siblings, and children) or second‐degree relatives (grandparents, grandchildren, uncles, aunts, nephews, nieces, and half‐siblings). Additionally, patients whose parents are first‐ or second‐degree cousins are identified as offspring of consanguineous parents. The circadian distributions of seizures were classified into three categories: those occurring solely while asleep (nocturnal), those exclusively manifesting during wakefulness (diurnal), and those occurring during both states (mixed). Additionally, diurnal seizures occurring within the first 2 h post‐arousal were specifically documented.

### 2.2 Inclusion/exclusion criteria

Consecutive patients enrolled in the NOEL trial between March 2010 and March 2020, meeting all the mandatory and none of the exclusionary ILAE criteria for the diagnosis of EGTCA^4^ were included in this study. Specifically, individuals with seizure onset between 5 and 40 years of age, who experienced one or more unprovoked GTC, with evidence of 3–5.5 Hz GSWD on EEG, and having at least a 2‐year follow‐up were included in this study. Patients with focal to bilateral tonic–clonic seizures, generalized myoclonic–tonic–clonic seizures, absence seizures, or myoclonic seizures were excluded as were patients whose EEGs showed focal or generalized slowing, focal spikes, or slow GSWD. Also excluded were patients with moderate or severe intellectual delay, progressive neurological conditions, or evidence of an epileptogenic lesion on brain MRI.

### 2.3 Variables

The following variables were collected at enrollment and follow‐up phone calls or clinic visits: demographics; disease characteristics (age at seizure onset, pretreatment seizure count, circadian distribution of seizures, seizure frequency, and response to ASM); epilepsy risk factors (family history of epilepsy, parental consanguinity, perinatal insult, febrile seizures, head trauma, and CNS infection); EEG finding and brain MRI results; and ASMs (dosage and posology, compliance, adverse events). If an ASM taper was implemented, we documented whether it was initiated by the patient or the treating physician, and the presence or absence of GSWD on the latest EEG before the taper.

### 2.4 Statistical analysis

Descriptive results were reported for the demographic and clinical characteristics. Cumulative time‐dependent probabilities of seizure recurrence for both ASM‐treated and untreated patients were calculated using Kaplan–Meier survival analysis. Similar analyses were conducted for the time‐dependent probability of seizure recurrence following ASM taper. Logistic regression analysis identified variables associated with seizure recurrence following ASM taper. Variables yielding *p*‐values <0.1 in univariate analysis were tested in a multivariate analysis with significance level of 0.05. Data were presented as odds ratios (OR) with 95% confidence intervals (CI).

### 2.5 Ethics approval and patient consent

This study was approved by the Institutional Review Board of the AUBMC, and all enrolled patients provided informed consent, signed either by themselves or one of their parents.

## 3 RESULTS

### 3.1 Patient characteristics

From March 2010 to March 2020, a total of 2345 adult and pediatric patients were enrolled in the NOEL trial. The distribution of those patients closely mirrored the geographical spread of the population across Lebanon's six administrative governorates. Among the enrolled individuals, 409 were diagnosed with either an IGE (286 patients) or a GGE (123 patients). Within the IGE cohort, 89 patients (59 men and 30 women) met the diagnostic criteria for EGTCA,^4^ constituting 31% of those diagnosed with an IGE. In addition, 59 patients only experienced unknown onset GTC seizures, with their epilepsy diagnosis remaining uncertain as both their initial and subsequent EEGs (when performed) yielded normal results. Noteworthy is that among these 59 patients, 50 experienced a single unprovoked GTC seizure without recurrence throughout the follow‐up period.

**FIGURE 1:**
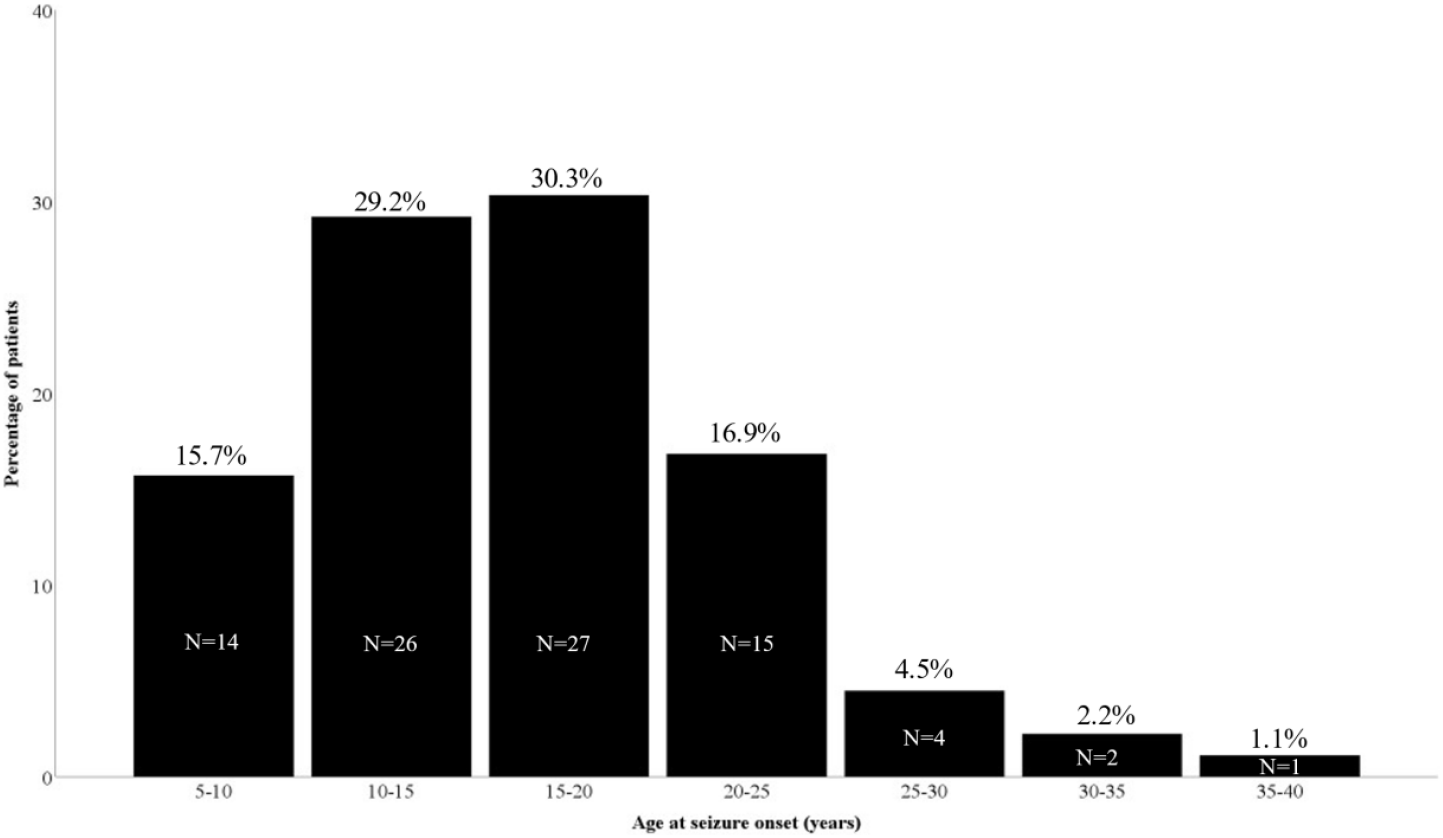
Distribution of patients according to age at seizure onset.

The mean age at seizure onset was 16.6 years (median 16.0 years; range: 5.4–38.3 years). Fourteen patients (15.7%) experienced their first seizure during the first decade, 53 (59.6%) during the second decade, 19 (21.3%) during the third decade, and three (3.4%) during the fourth decade. The patients' demographic and clinical characteristics are shown in Table 1, and their distribution according to age at seizure onset is illustrated in Figure 1. Regarding the circadian distribution of seizures, 53 patients (59.6%) exclusively experienced diurnal seizures, 11 (12.4%) exclusively experienced nocturnal seizures, and 25 (28.1%) experienced a combination of diurnal and nocturnal seizures. Among those with exclusively diurnal seizures, 27 experienced their seizures within 2 h of awakening. The mean follow‐up was 7.7 years (median 8.0 years; range: 2.0–14.7 years).

**TABLE 1:** Demographic characteristics and epilepsy risk factors of the study population.

Variable	
Mean age at seizure onset (years) ± SD	16.6 ± 6.3
Mean duration of follow‐up (years) ± SD	7.7 ± 2.9
	*N* (%)
Gender	
Male	59 (66.3%)
Female	30 (33.7%)
Circadian distribution of seizures	
Diurnal	53 (59.6%)
Nocturnal	11 (12.4%)
Mixed	25 (28.1%)
Number of epilepsy risk factors	
None	32 (36.0%)
1	44 (49.4%)
2	11 (12.4%)
≥3	2 (2.2%)
Type of epilepsy risk factor	
Family history of epilepsy (first degree)	12 (13.5%)
Consanguinity (first degree)	8 (9.0%)
Febrile seizures	9 (10.1%)
Perinatal insult	0
CNS infection	0

Abbreviations: CNS, central nervous system; SD, standard deviation.

Twelve patients (13.5%) reported a family history of seizures in a first‐degree relative, whereas 30 (33.7%) reported such a history in a second‐degree relative. The parents of eight patients (9.0%) were first‐degree cousins, whereas 12 (13.5%) were second‐degree cousins. Nine patients (10.1%) reported a history of febrile seizures. Four patients (4.5%) were diagnosed with mild developmental delay and five patients (5.6%) had concomitant psychiatric comorbidity, predominantly depression and/or anxiety disorder. Forty‐six patients (51.7%) were unable to identify a trigger for their seizures. Among the remaining 43 patients, the most frequent precipitating factors included sleep deprivation reported by 34 patients and severe stress reported by nine. At the initial visit, 56 patients (62.9%) presented with a single GTC, 30 (33.7%) experienced two GTCS, and three (3.4%) presented with a history of more than two GTCs.

### 3.2 Findings on initial EEGs

An average of 5.2 EEGs per patient were obtained throughout the follow‐up period. At the time of their initial EEG, 62 patients were untreated while 27 had started on an ASM. All initial EEGs included sleep recording. GSWD were observed in the initial EEG of 78 patients, while in 11 patients, GSWD were detected in subsequent EEGs. Among the 11 patients without epileptiform discharges on their initial EEG, GSWD were identified in eight patients during the second EEG, in two during the third, and in one patient during the fourth. No significant difference in GSWD detection frequency was observed between treated (22 out of 27; 81.5%) and untreated (56 out of 62; 90.3%) patients (chi‐squared; *p* = 0.2). A photoparoxysmal response was elicited on the initial EEG of 18 patients (20.2%) and in 31 patients (34.8%) when all follow‐up EEGs are considered.

### 3.3 Initial treatment with ASMs and seizure recurrence

Out of the 56 patients evaluated following a single GTC, 42 elected treatment with an ASM while 14 opted for lifestyle modifications alone. Among the 30 patients with two GTCs, 29 opted for ASM treatment while one chose lifestyle modifications. All three patients with more than two GTCs were prescribed an ASM.

Valproate was the most prescribed initial ASM for 58 patients (68.2%), followed by levetiracetam for 18 (21.2%), phenytoin for 4 (4.7%), lamotrigine for 3 (3.5%), and carbamazepine and topiramate for 1 patient each. Twenty‐two patients (25.9%) switched their initial ASM due to adverse events or lack of efficacy.

**FIGURE 2:**
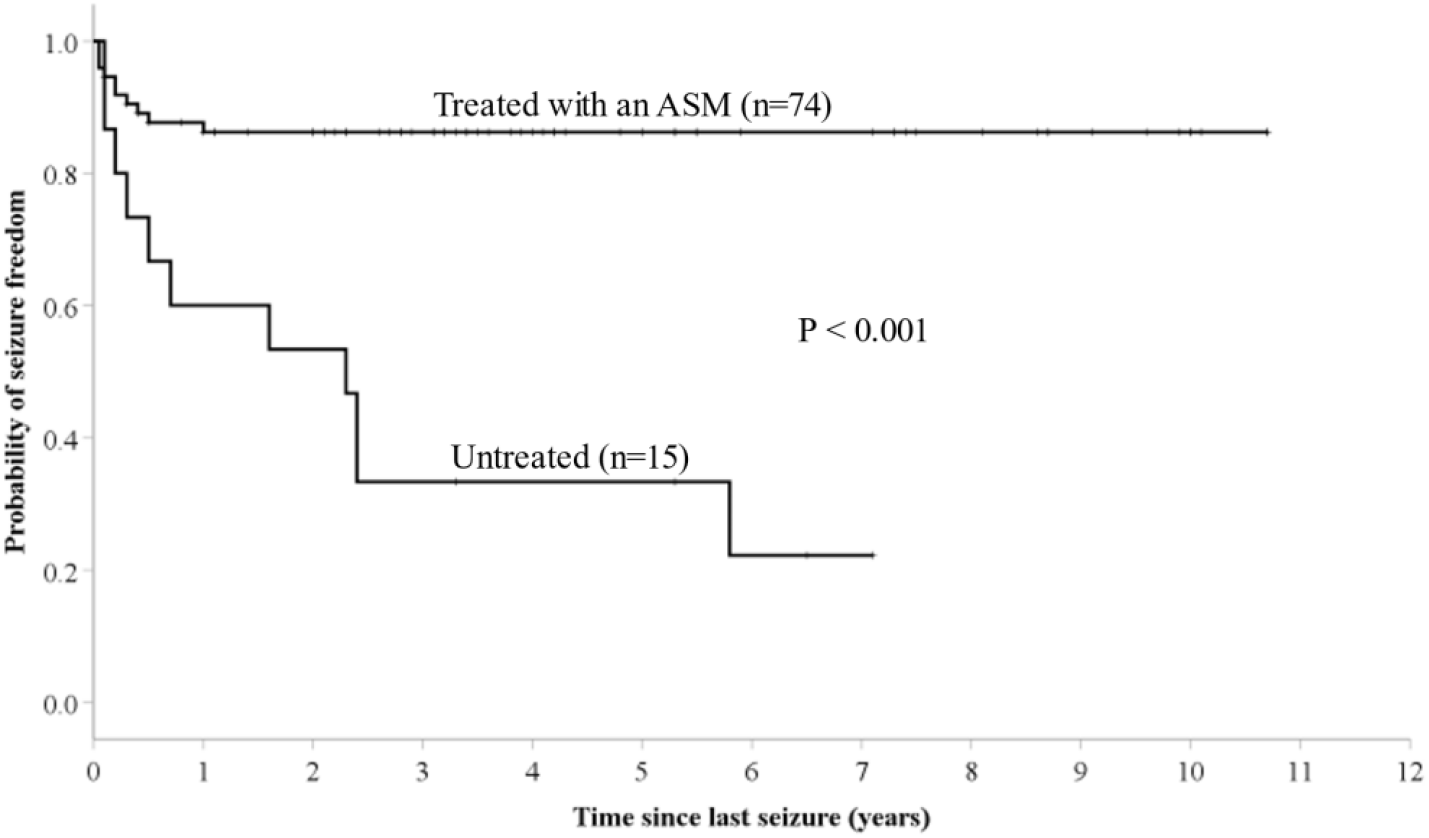
Kaplan–Meier plot of time to first seizure recurrence, stratified between treated and untreated groups.

A GTC recurred in 11/15 (73.3%) untreated patients compared with 10/74 (13.5%) treated patients (*p* < 0.00001). All recurrences exclusively consisted of GTC seizures. Kaplan–Meier survival curves showed a significant difference in time to recurrence between treated and untreated patients (Figure 2, *p* < 0.001). In the treated group, all recurrences occurred during the first year of treatment (Figure 2). In the untreated group, six patients (54.5%) recurred during the first year and 10 (90.9%) during the first 30 months, with one patient experiencing a recurrence nearly 6 years later (Figure 2). The difference in time to recurrence between the treated and untreated groups remained significant in the subgroups of children, adults, women, men, presence or absence of a family history, and with or without parental consanguinity (*p* < 0.05 for all subgroups).

Of the 14 patients who presented with a single GTC and opted not to initiate treatment, 11 (78.6%) experienced a recurrence and started on an ASM after the second seizure. The sole patient who elected against ASM treatment after experiencing two GTCs remained seizure‐free.

**FIGURE 3:**
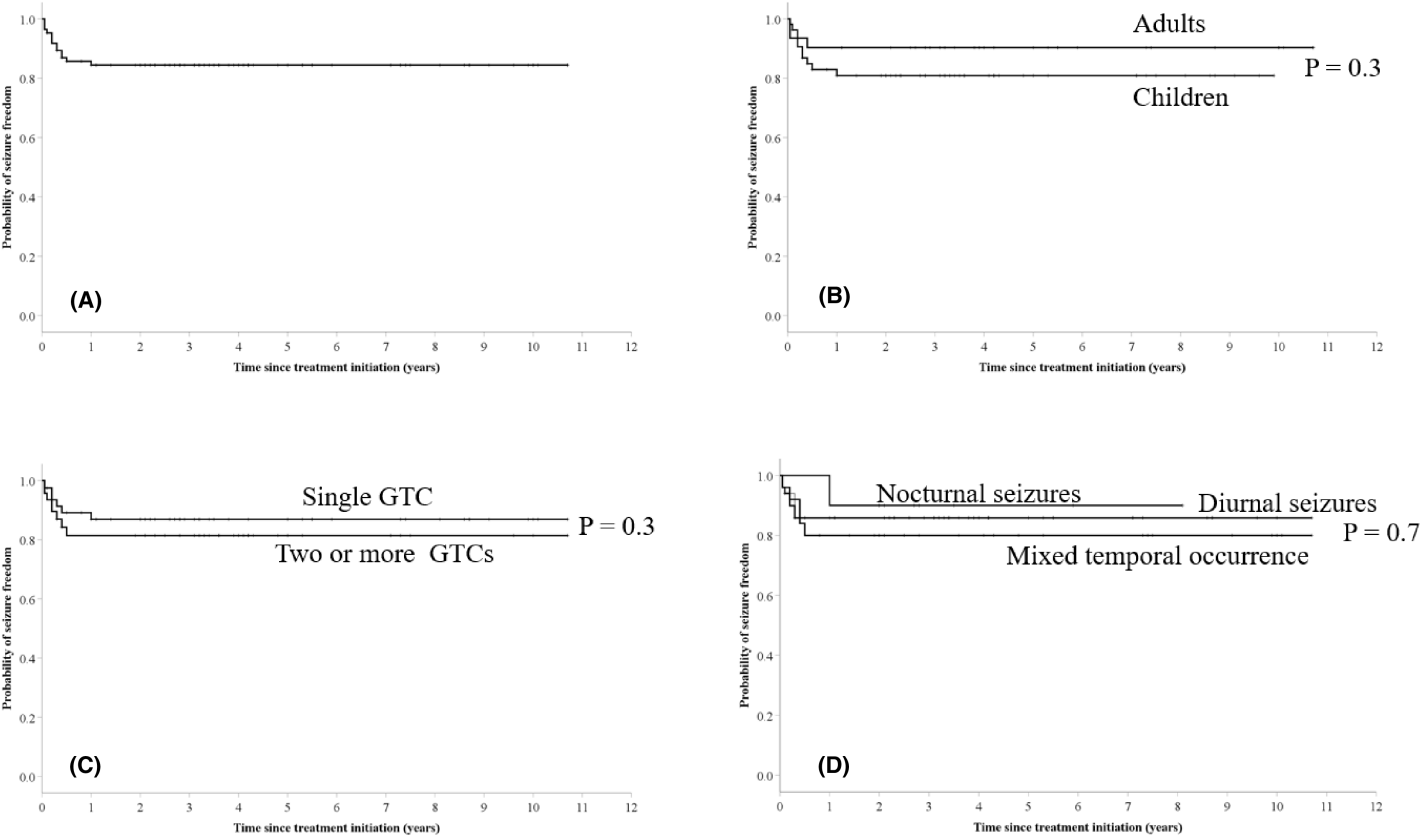
Kaplan–Meier plot of time to first seizure recurrence. (A) The group of 85 patients who were started on antiseizure medication (ASM) treatment at some point during their disease course. (B) Comparison of time to first seizure recurrence between adults (>18 years) and children. (C) Comparison of time to first seizure recurrence between patients who received ASM treatment following a single GTC and those treated after two or more GTCs. (D) Comparison of time to first seizure recurrence between patients who exclusively experienced diurnal seizures, exclusively experienced nocturnal seizures and those who experienced seizures with a mixed temporal occurrence.

Among the 85 patients who were treated at some point during their disease course, 13 (15.3%) experienced a recurrence (Figure 3A), with no significant difference in time to recurrence between subgroups of adults and children (Figure 3B), men and women, those treated after a single or multiple GTCs (Figure 3C), and according to the circadian distribution of seizures (Figure 3D).

The four untreated patients remained seizure‐free, with a mean follow‐up of 5.3 years (median 5.4 years; SD 1.8 years; range: 3.3–7.1 years).

### 3.4 First attempt at ASM discontinuation

ASM discontinuation was attempted in 50 patients after a mean treatment duration of 3.3 years (median 3.0 years, SD 2.3 years) with the majority having maintained a seizure‐free status for at least 2 years prior to taper initiation. The decision to discontinue treatment was made by a physician in 26 cases, and by patients in 24 instances. Physicians in our study typically considered ASM taper after at least a two‐year period of seizure remission, contingent on the latest EEG confirming the absence of GSWD, with a gradual taper over a two‐month period.

**FIGURE 4:**
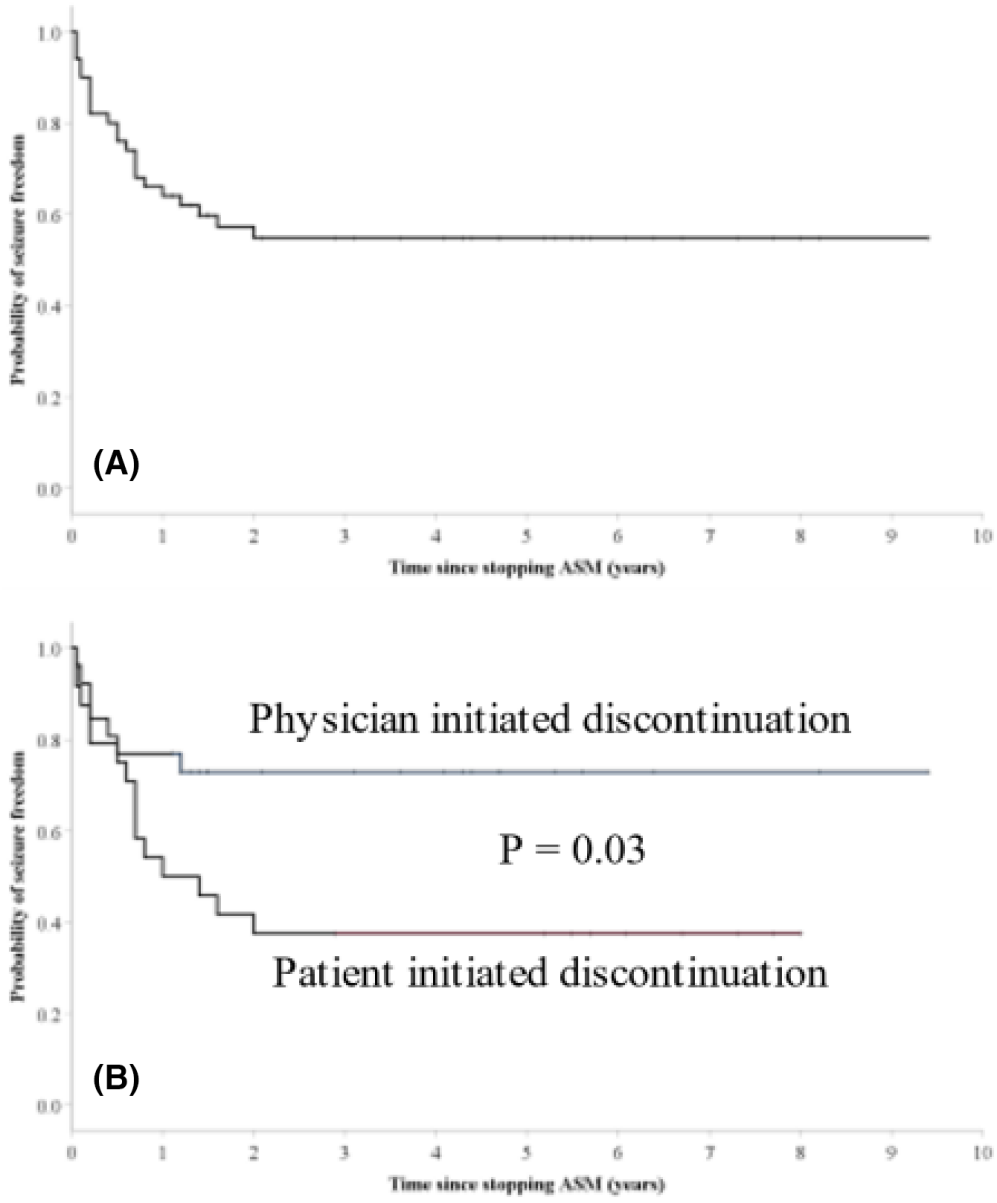
(A) Kaplan–Meier plot of time to first seizure recurrence following antiseizure medication (ASM) discontinuation. (B) Comparison of time to first seizure recurrence following ASM discontinuation between the group of physician‐initiated ASM taper and patient‐initiated ASM taper.

The Kaplan–Meier survival curve in Figure 4 illustrates time to recurrence. Of the 22 patients (44%) who experienced a recurrence, 18 did so within the first‐year post‐discontinuation and the rest between the first and second years. A significant difference in time to seizure recurrence was noted between physician‐initiated and patient‐initiated taper groups (Figure 4B).

ASM discontinuation was not attempted in 35 patients for reasons including seizure recurrence during treatment, persistence of GSWD on the EEG, or patient's unwillingness to risk seizure recurrence.

### 3.5 Predictive variables for seizure recurrence following ASM discontinuation

We assessed the association of several variables with the likelihood of seizure recurrence post‐ASM discontinuation. Univariate logistic regression identified four variables significantly associated with a higher frequency of recurrence (Table 2). They included whether the taper was physician‐ or patient‐initiated, presence or absence of GSWD on the latest EEG, presence or absence of a family history of epilepsy in a first‐degree relative, and circadian occurrence of seizures (Table 2). No significant associations were found with age at seizure onset, age at ASM discontinuation, gender, parental consanguinity, duration of seizure freedom on ASM, and number of seizures at time of discontinuation.

**TABLE 2:** Univariate and multivariate logistic regression analysis for seizure recurrence following ASM taper.

	Univariate analyses				Multivariate analyses
	Seizure recurrence *N* (%)		*p* value	Odds ratio (95% CI)	Odds ratio (95% CI)
	No	Yes			
Patient‐initiated	9 (37.5%)	15 (62.5%)	0.01	4.5 (1.4–15.0)	10.7 (1.7–65.4)
Physician‐initiated	19 (73.1%)	7 (26.9%)			
GSWD on EEG^a^	4 (30.8%)	9 (69.2%)	0.007	7.9 (1.8–34.9)	NS
No GSWD on EEG	21 (77.8%)	6 (22.2%)			
Mixed temporal seizure pattern	5 (33.3%)	10 (66.7%)	0.04	3.8 (1.1–13.8)	10.1 (1.7–59.2)
Diurnal or nocturnal only	23 (65.7%)	12 (34.3%)			
First‐degree family history					
Yes	1 (14.3%)	6 (85.7%)	0.04	10.1 (1.1–91.9)	NS
No	27 (62.8%)	16 (37.2%)			

^a^ Forty out of 50 patients had EEGs performed within 1 year prior to discontinuation.

Multivariate logistic regression (Table 2) identified patient‐initiated taper (OR = 10.7) and mixed circadian seizures (OR = 10.1) as the only independent predictors of higher likelihood of seizure recurrence after ASM discontinuation.

### 3.6 Second attempt at ASM discontinuation

A second attempt to discontinue treatment was undertaken for nine patients after an average treatment duration of 3.3 years following the initial failed attempt. This was successful for six patients, with a mean duration of seizure freedom post‐discontinuation of 1.9 years.

### 3.7 Final outcome at last follow‐up

At the last follow‐up, 51 patients (57.3%) were still receiving ASM, with a mean terminal seizure freedom of 3.6 years (median 2.5 years, SD 2.6 years, range: 0.2–9.7 years). Among these patients, only four experienced a terminal remission lasting less than 1 year. The majority of patients were on monotherapy, including 27 on valproate, 16 on levetiracetam, 3 on lamotrigine, 2 on topiramate, and 1 on brivaracetam, while two patients were on dual therapy with levetiracetam and valproate.

The remaining 38 patients (42.7%) were off ASM and remained seizure‐free off treatment for a mean duration of 4.4 years (median 4.4 years, SD 2.3 years, range: 0.8–9.4 years) and a mean terminal seizure freedom of 6.9 years (median 6.5 years, SD 2.5 years, range: 1.6–11.6 years).

## 4 DISCUSSION

This is the first comprehensive prospective study assessing demographics, electroclinical findings, treatment response, and feasibility of ASM discontinuation in a large cohort of patients newly diagnosed with EGTCA.

Our data indicate that EGTCA accounts for 31% of the IGE, a prevalence falling within the range reported by the ILAE and other studies.^4^, ^5^, ^6^ Most patients experience their first seizure during the second decade, with a mean age at seizure onset of 16.6 years, consistent with other studies.^4^, ^6^, ^7^, ^8^, ^9^, ^10^ Although the age at seizure onset in our study ranged from 5.4 to 38.3 years, it falls within the 5–40 years range recommended by the ILAE.^4^

Within our cohort, 47% had a family history of seizures in first or second‐degree relatives, a percentage falling within the 20%–58% range reported in previous studies on EGTCA.^7^, ^8^, ^10^, ^11^ This high prevalence is likely attributable to the genetic inheritance of this syndrome.^12^, ^13^ Nearly half of our patients reported seizure‐provoking factors, mostly sleep deprivation, a recognized trigger in EGTCA^7^, ^10^ and one of the most effective provocation methods during routine EEGs.^1^, ^14^

The syndrome of EGTCA was formerly known as epilepsy with GTC upon awakening.^1^, ^2^ In our study, only 30% of patients exclusively experienced seizures within 2 h upon waking, a finding that challenges the conventional view which posits that most seizures in this syndrome will occur shortly after awakening.^4^, ^8^ Our results indicate that seizures could manifest at any time of the day or night, with a tendency toward a mixed circadian pattern in 28% of patients. Those findings align with a recent study that reported the absence of clear‐cut circadian patterns in 43% of individuals with EGTCA.^10^

Our investigation unveils novel and previously unreported data on the yields of sequential EEGs in detecting GSWD in patients with new‐onset GTC seizures, subsequently diagnosed with EGTCA. The significance of these findings is underscored by the mandatory presence of GSWD for diagnosing EGTCA.^4^ We found that a 3‐h sleep‐deprived EEG is a valuable initial investigation, with GSWD documented in 88% of tracings, and with a slightly but not significantly lower detection rate in treated versus untreated patients. Although there is a lack of published data specific to EGTCA, GSWD were more generally reported to be present on the initial EEG of 30% to 80% of patients ultimately diagnosed with an IGE,^15^, ^16^ necessitating serial recordings for syndrome diagnosis clarification. This variability in detection rates can be attributed to various factors including the use of ASM at the time of EEG acquisition, timing of EEG relative to seizure occurrence, sleep deprivation, and EEG recording duration. Notably, a photoparoxysmal response was present in 20% of initial EEGs, and in 35% when considering all follow‐up recordings. Photosensitivity is common in EGTCA, with reported frequencies ranging from 13% to 62%.^10^, ^17^ Our results also emphasize the importance of repeating EEGs in patients without initial epileptiform discharges, as a second recording increases the GSWD detection rate to 96.6% with additional yields in subsequent tracings. It is however important to note that within our initial cohort, the diagnoses of some patients remained uncertain because they experienced unknown onset GTC seizures alongside normal EEG results. It is therefore conceivable that some of these patients could have been diagnosed with EGTCA had an ambulatory study with overnight recording been performed.

Our results underscore the importance of initiating prompt ASM treatment upon EGTCA diagnosis, given the high recurrence rate in untreated patients. Indeed, 73% of patients who solely opted for lifetime modifications experienced a seizure recurrence, compared with 14% of those treated with an ASM. This difference remained significant across subgroups, including children and adults, men and women, and regardless of familial history of epilepsy or parental consanguinity. Among the ASM‐treated group, all recurrences occurred during the first year of treatment. While most recurrences in untreated patients transpired during the initial year, a sizeable proportion occurred between 13 and 30 months with one patient experiencing a recurrence as late as 6 years after the initial GTC. Late recurrences may result from sporadic isolated GTCs triggered by factors such as sleep deprivation, stress, or alcohol intake.^18^ In the ASM‐treated group, no significant differences in recurrence rates were observed according to gender, age at seizure onset, or circadian pattern of seizures. Furthermore, there was no significant difference in relapse rates between patients treated after their first GTC and those initiated on treatment after more than one seizure. This finding aligns with the FIRST trial results, notwithstanding the inclusion of patients with focal to bilateral tonic–clonic seizures in that trial.^19^ Our findings differ from a previous study that suggested that a higher number of GTCs prior to initiating ASM increased the risk of drug resistance.^10^ However, it is important to note that the subgroup more predisposed to developing drug resistance in that study experienced at least weekly or monthly seizures before ASM initiation.^10^ In contrast, nearly all patients in our cohort were treated after experiencing their first or second seizure.

Most patients in our study responded well to treatment with an ASM, with only two individuals meeting the criteria for medical refractoriness.^20^ At the last follow‐up, all but two patients were successfully managed on a single ASM, achieving sustained terminal seizure freedom for over a year in most cases, findings consistent with the existing literature.^8^, ^9^, ^11^, ^21^ Despite concerns regarding valproate administration to women of childbearing age, this drug was the most prescribed initial ASM, a trend consistent with previous studies.^10^, ^21^, ^22^ This likely reflects the perceived efficacy of valproate in individuals with IGE as demonstrated in a randomized trial.^23^

Within our cohort, 50 out of 85 seizure‐free patients on ASM for an average of 3 years attempted discontinuation. Successful withdrawal was achieved in 34 (68%), with 28 remaining seizure‐free for over 4 years after the first attempt. The remaining six patients succeeded after their second attempt and maintained seizure freedom for a mean duration of 1.9 years off treatment. Literature on recurrences following ASM discontinuation in EGTCA is limited, with reported relapse rates ranging from 18% to 80%.^8^, ^10^, ^11^, ^21^, ^24^, ^25^ Discrepancies in these rates may be attributable to study design variations with most previous studies being retrospective, patient heterogeneity, disparities in timings of treatment initiation and discontinuation, sample sizes, patient or physician‐initiated withdrawal, and EEG findings before ASM tapering. Noteworthy is the temporal pattern of recurrences in our study, with the majority manifesting within the initial year post‐ASM withdrawal, aligning with previous studies conducted in patients with IGEs.^10^, ^21^, ^24^

In multivariate analysis, the only two variables that independently predicted a higher risk of recurrence after ASM discontinuation were patient‐initiated taper and a mixed circadian seizure occurrence. In fact, patients initiating their taper were nearly 11 times more likely to experience a relapse compared with those with a physician‐initiated taper, aligning with findings from a previous study.^21^ This disparity in recurrence rate can be explained by the practice among physicians in our study, who typically initiated ASM taper on patients who remained seizure‐free on treatment for at least 2 years and after documenting the absence of GSWD on the EEG. Indeed, we found that the presence of GSWD prior to taper was significantly associated with seizure recurrence in univariate but not in multivariate analysis, likely due to its consideration in physician‐initiated taper decisions. In a study conducted in patients with GGE, the presence of GSWD at the time of withdrawal was associated with a non‐significant increase in recurrences, likely due to the small sample size.^21^ Future studies with larger sample size should explore the predictive significance of GSWD prior to, during, and after ASM discontinuation in patients with EGTCA.

A previously unreported finding in our study is the 10‐fold higher seizure recurrence risk after ASM discontinuation in patients with mixed diurnal and nocturnal GTCs compared with those with exclusive diurnal or nocturnal seizures. A previous study reported that patients with seizures upon waking were less likely to be seizure‐free compared with those with random GTCs,^7^ findings that were subsequently refuted.^9^ It is worth noting that in both of those studies, seizures occurring in the late afternoon or evening hours of relaxation were included in their definition of “seizures upon awakening.”

First‐degree family history of epilepsy showed a higher recurrence risk on univariate not in multivariate analysis, consistent with recent metanalysis findings.^26^ Age at seizure onset and duration of seizure freedom on ASM were not significant predictors of recurrence in our study, differing from a prior study that only included adolescents with various IGE syndromes.^21^

Our findings suggest the need for individualized duration of treatment for EGTCA patients, challenging the conventional paradigm of lifelong ASM for all.^21^ Our data posit that following thorough consultation with both the patients and their families, consideration of ASM taper is appropriate for select patients, seizure‐free for two or more years, with a recent EEG showing no GSWD. Since most recurrences occur during the first‐year post‐ASM discontinuation, close monitoring and explicit instructions on seizure precautions and driving restrictions are crucial. It is also important to emphasize compliance, as our observations indicate a high relapse rate in patient‐initiated taper.

This study possesses several strengths, including a prospective design, selective inclusion of newly diagnosed patients, multicenter referral mirroring population distribution, strict adherence to ILAE criteria, extended follow‐up, and centralized, standardized evaluations. Some limitations must be acknowledged, with the most important being the non‐randomized ASM withdrawal selection, potentially influenced by the treating physician's judgment. Additionally, systematic acquisition of EEGs during and after ASM discontinuation were lacking, precluding evaluation of these variables as prognostic factors. Future investigations should explore the significance of GSWD reappearance on EEG during and after tapering in determining ASM withdrawal success.

In conclusion, our prospective study on newly diagnosed patients with EGTCA revealed a broad age range at seizure onset, primarily in the second decade of life. Seizures occurred diurnally and nocturnally, with 28% approximately one‐third of patients exhibiting a mixed circadian pattern. Our findings emphasized the importance of obtaining a sleep‐deprived EEG and the need for prompt treatment upon the diagnosis of EGTCA. Seizures were responsive to treatment with an ASM, with a minority satisfying the definition of drug resistance. Notably, lifelong treatment may not be imperative; patients who remained seizure‐free for at least 2 years, with the absence of GSWD on EEG, often maintained seizure freedom post‐ASM withdrawal, especially with physician‐initiated tapering.

## AUTHOR CONTRIBUTIONS

Fatima Jaafar substantially contributed to the acquisition and analysis of the data and drafting of the work; final approval of the version to be published; and agreement to be accountable for all aspects of the work. Jaafar Wazne, Ghassan Hmeimess, and AbdelRahman Shatila substantially contributed to the acquisition of the data and drafting of the work; final approval of the version to be published; and agreement to be accountable for all aspects of the work. Wassim Nasreddine substantially contributed to the acquisition, analysis, and interpretation of the data and drafting of the work; final approval of the version to be published; and agreement to be accountable for all aspects of the work. Ayman Beydoun substantial contributed to the acquisition of the data and drafting of the work; final approval of the version to be published; and agreement to be accountable for all aspects of the work. Ahmad Beydoun substantially contributed to the conception and design of the work; the acquisition, analysis, and interpretation of data for the work and drafting of the work; final approval of the version to be published; and agreement to be accountable for all aspects of the work.

## CONFLICT OF INTEREST STATEMENT

None of the authors has any conflict of interest to disclose. We confirm that we have read the Journal's position on issues involved in ethical publication and affirm that this report is consistent with those guidelines.

## References

[1] Janz D . Epilepsy with grand mal on awakening and sleep‐waking cycle. Clin Neurophysiol. 2000;111:S103–S110. 10996562 10.1016/s1388-2457(00)00409-0

[2] Proposal for revised classification of epilepsies and epileptic syndromes. Commission on classification and terminology of the international league against epilepsy. Epilepsia. 1989;30(4):389–399. 2502382 10.1111/j.1528-1157.1989.tb05316.x

[3] Engel J Jr . A proposed diagnostic scheme for people with epileptic seizures and with epilepsy: report of the ILAE task force on classification and terminology. Epilepsia. 2001;42(6):796–803. 11422340 10.1046/j.1528-1157.2001.10401.x

[4] Hirsch E , French J , Scheffer IE , Bogacz A , Alsaadi T , Sperling MR , et al. ILAE definition of the idiopathic generalized epilepsy syndromes: position statement by the ILAE task force on nosology and definitions. Epilepsia. 2022;63(6):1475–1499. 35503716 10.1111/epi.17236

[5] Mullins G , O'sullivan SS , Neligan A , McCarthy A , McNamara B , Galvin RJ , et al. A study of idiopathic generalised epilepsy in an Irish population. Seizure. 2007;16(3):204–210. 17223580 10.1016/j.seizure.2006.12.007

[6] Gesche J , Christensen J , Hjalgrim H , Rubboli G , Beier CP . Epidemiology and outcome of idiopathic generalized epilepsy in adults. Eur J Neurol. 2020;27(4):676–684. 31838768 10.1111/ene.14142

[7] Unterberger I , Trinka E , Luef G , Bauer G . Idiopathic generalized epilepsies with pure grand mal: clinical data and genetics. Epilepsy Res. 2001;44(1):19–25. 11255069 10.1016/s0920-1211(00)00210-2

[8] Holtkamp M , Kowski AB , Merkle H , Janz D . Long‐term outcome in epilepsy with grand mal on awakening: forty years of follow‐up. Ann Neurol. 2014;75(2):298–302. 24395517 10.1002/ana.24103

[9] Vorderwülbecke BJ , Kowski AB , Kirschbaum A , Merkle H , Senf P , Janz D , et al. Long‐term outcome in adolescent‐onset generalized genetic epilepsies. Epilepsia. 2017;58(7):1244–1250. 28464258 10.1111/epi.13761

[10] Cerulli Irelli E , Gesche J , Schlabitz S , Fortunato F , Catania C , Morano A , et al. Epilepsy with generalized tonic‐clonic seizures alone: electroclinical features and prognostic patterns. Epilepsia. 2023;65:84–94. 37872695 10.1111/epi.17809

[11] Camfield P , Camfield C . Idiopathic generalized epilepsy with generalized tonic‐clonic seizures (IGE‐GTC): a population‐based cohort with> 20 year follow up for medical and social outcome. Epilepsy Behav. 2010;18(1–2):61–63. 20471324 10.1016/j.yebeh.2010.02.014

[12] Tan NC , Berkovic SF . The epilepsy genetic association database (epiGAD): analysis of 165 genetic association studies, 1996–2008. Epilepsia. 2010;51(4):686–689. 20074235 10.1111/j.1528-1167.2009.02423.x

[13] Weber YG , Lerche H . Genetic mechanisms in idiopathic epilepsies. Dev Med Child Neurol. 2008;50(9):648–654. 18754913 10.1111/j.1469-8749.2008.03058.x

[14] Halász P , Filakovszky J , Vargha A , Bagdy G . Effect of sleep deprivation on spike‐wave discharges in idiopathic generalised epilepsy: a 4× 24 h continuous long term EEG monitoring study. Epilepsy Res. 2002;51(1–2):123–132. 12350388 10.1016/s0920-1211(02)00123-7

[15] Owolabi LF , Sale S , Owolabi SD , Nalado A , Umar M , Taura AA . Electroencephalography abnormalities in generalized epilepsy and their predictors: a multicenter experience. Ann Afr Med. 2018;17(2):64–69. 29536959 10.4103/aam.aam_2_17 PMC5875121

[16] Betting LE , Mory SB , Lopes‐Cendes I , Li LM , Guerreiro MM , Guerreiro CAM , et al. EEG features in idiopathic generalized epilepsy: clues to diagnosis. Epilepsia. 2006;47(3):523–528. 16529616 10.1111/j.1528-1167.2006.00462.x

[17] Covanis A . Photosensitivity in idiopathic generalized epilepsies. Epilepsia. 2005;46:67–72. 16302877 10.1111/j.1528-1167.2005.00315.x

[18] Sokic D , Ristic AJ , Vojvodic N , Jankovic S , Sindjelic AR . Frequency, causes and phenomenology of late seizure recurrence in patients with juvenile myoclonic epilepsy after a long period of remission. Seizure. 2007;16(6):533–537. 17574449 10.1016/j.seizure.2007.03.012

[19] Musicco M , Beghi E , Solari A , Viani F , First Seizure Trial Group (FIRST Group) . Treatment of first tonic‐clonic seizure does not improve the prognosis of epilepsy. Neurology. 1997;49(4):991–998. 9339678 10.1212/wnl.49.4.991

[20] Kwan P , Arzimanoglou A , Berg AT , Brodie MJ , Allen Hauser W , Mathern G , et al. Definition of drug resistant epilepsy: consensus proposal by the ad hoc task force of the ILAE commission on therapeutic strategies. Epilepsia. 2010;51(6):1069–1077. 19889013 10.1111/j.1528-1167.2009.02397.x

[21] Komatsubara T , Kobayashi Y , Hiraiwa A , Magara S , Hojo M , Ono T , et al. Recurrence rates and risk factors for seizure recurrence following antiseizure medication withdrawal in adolescent patients with genetic generalized epilepsy. Epilepsia Open. 2022;7(2):332–343. 35445562 10.1002/epi4.12603 PMC9159251

[22] Jomaa N , Nasreddine W , Hmeimess G , Beaini S , Beydoun A , Hotait M , et al. Risk of recurrence in patients with an unprovoked tonic‐clonic seizure and generalized epileptiform discharges on EEG. Epilepsia. 2023;64:2153–2161. 37264785 10.1111/epi.17671

[23] Marson A , Burnside G , Appleton R , Smith D , Leach JP , Sills G , et al. The SANAD II study of the effectiveness and cost‐effectiveness of valproate versus levetiracetam for newly diagnosed generalised and unclassifiable epilepsy: an open‐label, non‐inferiority, multicentre, phase 4, randomised controlled trial. Lancet. 2021;397(10282):1375–1386. 33838758 10.1016/S0140-6736(21)00246-4 PMC8047813

[24] Pavlović M , Jović N , Pekmezović T . Antiepileptic drugs withdrawal in patients with idiopathic generalized epilepsy. Seizure. 2011;20(7):520–525. 21493107 10.1016/j.seizure.2011.03.007

[25] Vorderwülbecke BJ , Kirschbaum A , Merkle H , Senf P , Holtkamp M . Discontinuing antiepileptic drugs in long‐standing idiopathic generalised epilepsy. J Neurol. 2019;266:2554–2559. 31267208 10.1007/s00415-019-09457-z

[26] Jiang T , Zhang X , Zhang M , Liu M , Zhu H , Sun Y . Drug‐resistant idiopathic generalized epilepsy: a meta‐analysis of prevalence and risk factors. Epilepsy Behav. 2023;146:109364. 37523796 10.1016/j.yebeh.2023.109364

